# Tobacco Use, Experiences and Knowledge Among Indigenous Mexican Agricultural Workers

**DOI:** 10.1007/s10903-025-01840-5

**Published:** 2025-12-25

**Authors:** Alison K. Herrmann, Genevieve Flores-Haro, Barbara Berman, Alison M. Elliott, Maritza Lopez, L. Cindy Chang, Norma Gonzalez, Catherine M. Crespi, Michael K. Ong, Arcenio Lopez, Roshan Bastani

**Affiliations:** 1 UCLA Kaiser Permanente Center for Health Equity; UCLA Fielding School of Public Health, University of California, Los Angeles, Los Angeles, United States; 2 Jonsson Comprehensive Cancer Center, University of California, Los Angeles, Los Angeles, United States; 3 Mixteco Indígena Community Organizing Project, Oxnard, United States; 4 School of Medicine, University of California, Los Angeles, Los Angeles, United States; 5 VA Greater Los Angeles Healthcare System, Los Angeles, United States

**Keywords:** Tobacco, E-cigarettes, Indigenous mexican, Immigrant, Survey

## Abstract

The unique culture and languages of Indigenous Mexican immigrants to the United States, a substantial portion of the nation’s agricultural workforce, has limited participation of this population in tobacco and other health related research. Our established community-academic team [Mixteco Indigena Community Organizing Project (MICOP) and UCLA] developed and conducted a community survey (*n* = 200) from February – October 2024, in Spanish (*n* = 145), English (*n* = 10) or Mixteco (*n* = 45), aimed to understand tobacco use and knowledge among adults in this population. Participants (mean age 26 years; 71% married; 48% female; 43% *≤* elementary education) reported considerable tobacco use and second-hand exposure. Results illustrate gaps in knowledge regarding tobacco’s health harms and limited experience with tobacco prevention or control programming. Our findings suggest that prevention programs may provide greatest benefits for recent immigrants who rely exclusively on their native Indigenous language, and that cessation resources may be most needed by community members who have been in the US longer, have some Spanish proficiency and greater exposure to industry marketing. This work adds to the limited literature regarding this marginalized population’s health needs and will serve as a guide for our ongoing community-academic efforts to develop, implement, evaluate and disseminate culturally and linguistically tailored tobacco prevention and control programming for the Indigenous Mexican agricultural worker community in California and beyond.

## Background

We report here on first steps in a research program aimed at addressing a significant gap in tobacco prevention and control efforts in California and the United States: the lack of adequate culturally and linguistically tailored tobacco prevention and control programming for the Indigenous Mexican farmworker community. Despite significant accomplishments in reducing tobacco use overall, the growth of newer tobacco products and persistent racial/ethnic and socio-economic disparities in use underscore that tobacco use remains an important public health concern. And yet, with respect to some populations, this problem is not well understood. For example, the National Farmworker Health Survey (NAWS), the most comprehensive source of data on U.S. agricultural workers with data collected annually since 1985 [1], has not included a question regarding tobacco use since 2004, and never included questions regarding second-hand smoke exposure or more novel product use. Identifying and addressing gaps in state and national research and programmatic efforts is critical to effectively mitigating this problem, countering tobacco industry influence, and protecting population health.

California’s agricultural workers, over 90% of whom are Mexican immigrants, are among the state’s most socio-economically challenged and historically underserved groups [2–7], experiencing long working hours, extreme poverty, limited formal education, and discrimination. On average laborers in this community earn less than $20,000 per year for a family of five, and a majority live in overcrowded housing, often with multiple families sharing a small apartment [8–10]. Immigration status and lack of health insurance contribute to community health risks and pose significant barriers to receipt of accurate health information and quality health services. Although the peer-reviewed literature is fairly limited regarding this population’s health, heightened cancer risks, chronic disease, obesity and substance use are widely recognized [11–15]. So too is an increased risk of respiratory illness and disease, in part due to exposure to environmental toxins in the work environment [16–23]. For the subgroup of agricultural workers who identify as Indigenous Mexicans, many of whom speak their native, primarily oral, language (e.g., Mixteco) and lack fluency in Spanish or English, these health risks, and barriers to tobacco and other health-related research and information are particularly acute. Recent research reveals a complex relationship between Indigenous identity among agricultural workers, documentation and insurance status and chronic health conditions [24–27].

In routine interactions with community members, staff and community health workers at Mixteco Indigena Community Organizing Project (MICOP; Mixteco.org), the first organization formed specifically to address the needs of Indigenous Mexican immigrants in California’s Central Coast, recognized gaps in community members’ tobacco-related knowledge and that few had experienced tobacco prevention programming. They also gained insight into tobacco use patterns and became aware of social norms precluding enforcement of non-smoking rules. Frequent second-hand smoke exposures described in these conversations are consistent with population-level data regarding non-smoking Latinos in California, especially those dwelling in multi-unit housing [28, 29]. In 2024 the agency partnered with researchers at UCLA to conduct a survey among community-dwelling adults aimed at systematically gathering information regarding tobacco use, knowledge, second-hand smoke exposure, and unmet programming needs. This research is consistent with MICOP’s over twenty-year commitment to supporting, organizing and empowering the Indigenous Mexican Community in California’s Central Coast, a region that is home to over 45,000 of the estimated 170,000 Indigenous Mexican immigrants in California, a majority of whom identify as Mixteco [8]– [9]. MICOP’s partnership in this project reflects the organizational commitment to addressing the Indigenous community’s most pressing social and health related needs.

## Conceptual Framework

The theoretical perspective of the Multi-Level Health Outcomes Framework (MHOF;, (previously the Health Behavior Framework), developed by Bastani and colleagues at UCLA [30–36], guided the study, including all procedures and development of data collection instruments. The MHOF has been used by the UCLA researchers and others in a range of studies targeting diverse ethnic, socio-cultural and linguistic groups in varied contexts, including community agencies [33, 37–40].

**Fig. 1:**
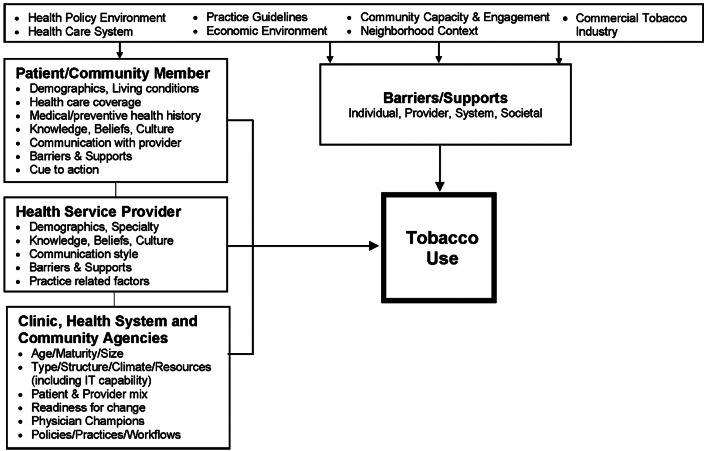
Multi-level health outcomes framework

The framework represents a synthesis of conceptual formulations in the area of health behavior and outcomes [41–48]. It takes a socio-ecological perspective [49], considering factors at patient/community member, provider and health care setting levels. It also recognizes the broader societal and community context within which health behaviors and health outcomes are embedded. Throughout this study and program of research, we focused primarily on community member characteristics, barriers and supports for tobacco product use, and the larger environmental context. Figure 1 provides a basic presentation of the MHOF, adapted for this study.

## Methods

### Community Advisory Committee and Study Staff Training

All aspects of this study were guided by a ten-member Community Advisory Committee (CAC), all of whom identified as Indigenous. CAC input was critical to survey development, and particularly finalization of survey items with consistent meaning in Spanish and Mixteco.

Two MICOP staff completed a two-day (8 h total) training in human subjects’ research and study-specific procedures (recruitment, consent, survey administration, data entry) provided by UCLA team members. Following this training, study staff practiced use of all study instruments and survey administration with CAC members, in Spanish and Mixteco. Notes and audio recordings were developed for study staff to reference as needed given Mixteco is a primarily oral language and all study instruments were written in Spanish and English.

### Participants

Following completion of the training, two MICOP staff members approached community members in public spaces (parks, community-based organizations, agricultural fields) to invite survey participation, describing the survey’s content, length and purpose. The survey was also advertised via MICOP’s radio station and social media channels, to increase awareness of community recruitment; interested individuals could also contact MICOP office in person or by phone. Participants (*n* = 200) identified as Indigenous Mexican, were aged 18–50 years, and spoke either Spanish or Mixteco, the most commonly spoken Indigenous language in the community.

### Data Collection

Between February – October 2024 surveys were administered by MICOP staff at the time of recruitment or a later scheduled date/time at MICOP’s office or in a local park, in Spanish (*n* = 145), English (*n* = 10) or Mixteco (*n* = 45). Resource limitations precluded inclusion of additional Indigenous languages. Participant responses were entered into a Qualtrics database, in real-time in Spanish, given Mixteco is a primarily oral language. Verbal consent was obtained from participants immediately prior to survey completion. Participants received a $50 gift card to a local grocery store.

### Measures

Survey items (see Appendix) were adapted from existing instruments including the National Adult Tobacco Survey, CDC’s Behavioral Risk Factor Surveillance System (BRFSS), WHO’s Global Adult Tobacco Survey and instruments used in our own prior work [50–53]. Draft items were translated, via translation, back translation, and cross-language equivalency procedures used by the team in prior work [37, 54–56]. Items were pilot tested among community advisors and MICOP staff members fluent in the Indigenous languages and culture of this community, to obtain feedback regarding ease of understanding and guidance in translation and adaptation of survey instruments into multiple Mixteco variants. The final instrument included 110 items. Questions assessed use, attitudes, knowledge, and community norms regarding tobacco, vaping and cannabis. To assess tobacco use, interviewers showed participants photos of 9 tobacco products (cigarettes, e-cigarettes/vapes, hookah, cigars, cigarillos, loose tobacco and rolling papers, nicotine pouches, pipes, chewing tobacco). Participants indicated which products they had tried in response to the item *“Have you ever tried any of these products?”* Those who responded yes were asked to indicate which products. For each product tried, participants were then asked *“Are you still using [tobacco product]?”* Those who responded yes were then asked *“About how many days a week do you normally use [tobacco product]?”* We categorized all participants who reported that they are still using one or more of the 9 tobacco products as current users. Similarly, to assess use among close friends, interviewers referenced the photos and asked *“Do any of your close friends use any of these products?”* Participants were able to select all that applied and those who reported that any close friends used any products were included in the exposed group. We report here on findings regarding tobacco use and vaping.

### Analysis

Analyses were primarily descriptive, including categories of tobacco product use, related attitudes, knowledge, exposure, and demographic characteristics. Analytic categories also describe exposure to tobacco marketing, health information, smoke-free policies, and prevention/control programming. To understand variations within the Indigenous population, stratified analyses were conducted by population group and language use. To further investigate differences between individuals who speak exclusively Mixteco versus those who speak any Spanish, bivariate comparisons (two-sample t-tests and chi-square tests) were conducted. We characterized those who completed the survey in Mixteco and indicated that they speak only Mixteco at home as monolingual Mixteco speakers (*n* = 37). Participants who completed the survey in Spanish (*n* = 145), who competed the survey in Mixteco yet indicated they speak some Spanish at home (*n* = 8), and who completed the survey in English and indicated they speak Spanish at home (*n* = 10) were characterized as those who speak any Spanish (*n* = 163). We conducted additional analyses by participants’ survey language (Spanish, English, Mixteco) and tobacco use (current users vs. non-users) and report significant results within the text. Statistical significance was assessed at the 0.05 level for all statistical tests. All statistical analyses were conducted using SAS version 9.4 for Windows (SAS Institute, 2023). All activities were reviewed and approved by the UCLA Office of Human Research Protection Program (OHRPP; IRB #22–000643).

## Results

### Demographics

Table 1 presents sample demographic, housing and employment characteristics stratified by language use (monolingual Mixteco vs. any Spanish) and overall. On average, participants were 26 years old (SD = 6.7; data not shown), most were married (71%) and approximately half were female (48%). The majority indicated that they had one or more children (71%), with nearly one-fourth (23%) reporting three or more children (data not shown). Nearly three-quarters of participants (73%) reported working in agriculture. The majority of participants were born in Mexico (89%; data not shown) and 11% were born in the U.S.

Overall, household sizes were large; 18% reported living with two to four other families and over one-quarter (26%) with 7–15 other people (data not shown). A majority of participants reported living in a rented room within an apartment (57%) or in a rented garage space (4%) (data not shown). Only one participant reported owning their home.

**Table 1:** Demographics Indigenous agricultural workers (*N* = 200), comparing monoligual Mixteco speakers and those who speak any Spanish

	Monolingual Mixteco Speakers (*n* = 37) *n* (%)	Those who Speak any Spanish (*n* = 163) *n* (%)	Total Sample *N* = 200 *n* (%)	*P*-values
**Age**				0.7885
18–20	7 (19)	27 (17)	34 (17)	
21–30	24 (65)	100 (62)	124 (63)	
> 30	6 (16)	34 (21)	40 (20)	
**Female**	22 (59)	74 (45)	96 (48)	0.1222
**Married**	31 (84)	111 (68)	142 (71)	0.0709
**Time in US**				0.0020
Born in US	3 (8)	19 (12)	22 (11)	
Pre 2020	9 (24)	84 (52)	93 (47)	
2020 or later	25 (68)	58 (36)	83 (42)	
**≤** **Elementary Education**	29 (78)	56 (35)	85 (43)	< 0.0001
**Household Size** *mean (sd)*	7 (2.6)	6 (2.8)	6 (2.8)	0.1128
**Families in Household**	1.9(0.6)	1.8 (0.9)	1.8 (0.8)	0.4711
**Housing Status~**				0.2704
Rent a room	24 (65)	89 (55)	113 (57)	
Rent an apartment/house	13 (35)	73 (45)	86 (43)	
**Employment**				0.0015
Agriculture	28 (76)	117 (72)	145 (73)	
Other	1 (3)	35 (21)	36 (18)	
No work outside home	8 (22)	11 (7)	19 (10)	

~ One Spanish speaking home owner excluded

Significant differences were found between monolingual Mixteco speakers and those who speak any Spanish, including regarding time in the U.S. and education. Among the 89% of participants who immigrated to the U.S. from Mexico, nearly half did so in 2020 (42%). Monolingual Mixteco speakers were more recent immigrants, as compared with those who spoke any Spanish (68% arrived to the U.S. in 2020 or later, vs. 36% of Spanish speakers, *p* =.0020). Overall, formal education was limited; 78% of monolingual Mixteco speakers and 34% of those who spoke any Spanish reported completing elementary school or less. Nearly one-fifth of participants overall had not completed elementary school; 54% among monolingual Mixteco speakers (data not shown). In comparing data by participants’ survey language, significant differences were observed with regard to time in the U.S., with 60% of those who completed the survey in Mixteco arriving in 2020 or later, as compared with 39% of those who responded in Spanish and none of those who responded in English (*p* <.00001). Educational attainment also varied by survey language, with 82% of those who responded in Mixteco completing elementary school or less, compared with 33% of Spanish language respondents and none of the English respondents (*p* <.0001).

### Tobacco Related Characteristics

Table 2 presents tobacco and e-cigarette use, second-hand exposure, exposure to marketing and other messaging and tobacco-related knowledge for the sample overall and stratified by language usage. Most survey items distinguished vapes from other tobacco products and data are reported accordingly. Significant differences were observed between monolingual Mixteco speakers and those who spoke any Spanish in all variables aside from current use, second-hand exposure in the home from outside or from someone who lives with you and ever having been asked about use by a doctor.

Ever Use: Nearly half of participants had *ever used* any tobacco product (47%; 19% monolingual Mixteco speakers vs. 53% those who spoke any Spanish, *p* =.0002) with most respondents who ever used one or more products reporting use of cigarettes (44%). A smaller proportion of participants reported ever using vapes (9%), cigars (8%), cigarillos (5%), pipes (3%) or chewing tobacco (1%); one participant reported having tried hookah and another oral nicotine pouches. Most who reported cigarette use did so exclusively (*n* = 58) whereas among those who vaped multiple product use was more common; only 2 participants reported vaping exclusively. Ever use varied similarly by survey language, with 22% of Mixteco respondents reporting ever using tobacco products, compared with 56% of Spanish respondents and 30% of English respondents (*p* =.0002).

Current Use: *Current use* was reported by 15% of participants (5% monolingual Mixteco speakers vs. 17% those who spoke any Spanish, NS). Current use of cigarettes was most common (13%) followed by vapes (3%), and pipes (2%). One respondent reported currently using chewing tobacco and another oral nicotine pouches.

In analyses comparing current users and non-users (data not shown), significant differences were observed such that current users were more likely than non-users to be older (3 vs. 20% 18–20 yrs.; 47 vs. 65% 21–30 yrs; 50 vs. 15% >30 yrs, *p* <.0001), male (86 vs. 46%, *p* <.0001), and have lived in the U.S. longer (3 vs. 12% born in the US; 70 vs. 42% arrived before 2020; 27 vs. 44% arrived in 2020 or later, *p* =.0194).

Second-hand exposure: Over one-third of respondents (36%) indicated that close friends or family use tobacco products. This was most pronounced among those who spoke any Spanish (5% monolingual Mixteco speakers vs. 43% of those who spoke any Spanish, *p* <.0001). Overall, 14% of respondents reported living with someone who uses tobacco. While only four respondents said that use is permitted inside their living space, 88% of participants indicated that smoking is allowed in the building where they live (data not shown). Overall, one-fourth of respondents (26%) indicated tobacco smoke regularly enters their home from somewhere in or around the building. One-third of respondents (33%) reported routinely riding in a vehicle where someone else is smoking tobacco, with 21% reporting routine exposure to vaping in a vehicle. Such exposures were significantly greater among those who spoke any Spanish than monolingual Mixteco speakers (*p* <.01). Use by close friends also varied by survey language such that 7% of Mixteco respondents, 46% of Spanish respondents and 30% of English respondents reported having close friends who currently use tobacco products (*p* <.0001). Current users were more likely than non-users to report having close friends that use tobacco products (60 vs. 32%, *p* =.0040).

**Table 2:** Tobacco Use, Second-Hand exposure, exposure to tobacco messaging and tobacco related knowledge among Indigenous Mexican agricultural workers (*N* = 200), comparing monolingual Mixteco speakers and those who speak any Spanish

	Monolingual Mixteco Speakers (*n* = 37) *n* (%)	Those who speak any Spanish (*n* = 163) *n* (%)	Total Sample *N* = 200 *n* (%)	*P*-values
***Tobacco Use*** *(Any tobacco product*,* including vapes)*				
Ever Use	7 (19)	87 (53)	94 (47)	0.0002
Current Use	2 (5)	28 (17)	30 (15)	0.0702
***Second-Hand Exposure***				
**Living Space via outside^**	6 (16)	46 (28)	52 (26)	0.1329
**Work~ (among those working, n = 181)**	0 (0)	36 (24)	36 (20)	0.0034
**Car**				
Tobacco	5 (14)	60 (37)	65 (33)	0.0063
Vaping	1 (3)	41 (25)	42 (21)	0.0025
**Community**				
Tobacco	30 (81)	105 (64)	135 (68)	0.0507
Vaping	9 (26)	79 (48)	88 (44)	0.0140
**Family/Friends Use** *(any tobacco/vapes)*				
Someone who lives with you	4 (11)	24 (15)	28 (14)	0.5357
Close Friends	2 (5)	70 (43)	72 (36)	< 0.0001
***Exposure to Tobacco Messaging***				
**Industry Marketing**	13 (35)	131 (80)	144 (72)	< 0.0001
Prior **Programming**	1 (3)	35 (21)	36 (18)	0.0073
Ever asked about use by a **doctor**	30 (81)	127 (78)	157 (79)	0.6721
***Knowledge***				
**Products are addictive**				
Tobacco	21 (57)	143 (88)	164 (82)	< 0.0001
Vapes	16 (43)	127 (78)	143 (72)	< 0.0001
**Products are moderately or very harmful**				
Tobacco	32 (86)	156 (96)	188 (94)	0.0330
Vapes	25 (68)	148 (91)	173 (87)	0.0002

^ Specific to tobacco smoke; ~ References exposure to tobacco or cannabis at work

Regarding workplace and community exposure (data not shown), 25% of participants employed outside the home (*n* = 181) reported smoking is allowed, or the no smoking policy is not enforced, in their workplace, with 20% of participants reporting second-hand exposure to tobacco at work. Among this group, exposures were reported to cigarettes and other combustible tobacco products (*n* = 33), and to vaping (*n* = 11). Considerable community exposure was also reported (68% to tobacco and 44% to vaping), on sidewalks (cigarettes, *n* = 84; vapes, *n* = 46), parks or beaches (cigarettes = 55; vapes, *n* = 23) and at family events or house parties (tobacco, *n* = 27; vaping, *n* = 13). Over one-third of participants indicated they would not ask others to refrain from smoking cigarettes (35%) or vaping (37%) in their presence even if the smoke or vapor bothered them (data not shown). Current users were more likely than non-users to report exposure to vaping in the community (70 vs. 39%, *p* =.0027) but did not otherwise differ in terms of workplace and community exposures.

Exposure to Tobacco Marketing: Most participants (72%) indicated they had seen advertisements for cigarettes or vapes, most frequently on posters at convenience stores (*n* = 86), social media (*n* = 66), billboards (*n* = 25) or media websites (Spanish news site such as Telemundo; *n* = 21) (data not shown). Such exposure was far greater among those who spoke any Spanish as compared with monolingual Mixteco speakers (80% vs. 35%, *p* <.0001). Exposure to marketing also varied significantly by survey language with 40% of Mixteco respondents reporting such exposures, compared with 81% of Spanish respondents and 90% of English respondents (*p* <.0001). Marketing exposure did not vary significantly between current users and non-users, however there was a trend for current users to have greater exposure to industry marketing (87 vs. 69%, *p* =.0757).

Prevention/Control Program Experience: Participants reported few prior opportunities to learn about the health consequences of tobacco use. Only 18% of participants reported awareness of community tobacco prevention or cessation services. Among monolingual Mixteco speakers, only one participant reported such awareness; an additional participant who took the survey in Mixteco but also spoke some Spanish reported awareness of these resources as well. Over a fifth of participants (21%) shared that a doctor had never asked if they use tobacco products. No differences in prior tobacco prevention and control program experience were observed between current users and non-users.

Knowledge: Addictive nature of products: Most participants (82%) indicated that cigarettes and other tobacco products are addictive. Fewer respondents (72%) indicated that vaping is addictive. Among monolingual Mixteco speakers, however, only 57% were aware that cigarettes are addictive (vs. 88% among Spanish speakers, *p* <.0001) and 43% indicated that vaping is addictive (vs. 78% among Spanish speakers, *p* <.0001). Those who completed the survey in Mixteco were least likely to know that tobacco is addictive (64% vs. 87% of Spanish respondents and 90% of English respondents; *p* =.0023) or that vapes are addictive (49% vs. 77% of Spanish respondents and 90% of English respondents; *p* =.0005). No differences in knowledge were observed between current users and non-users.

Health harms: Although most participants (94%) described cigarettes and other tobacco products as harmful to health, fewer recognized the health harms of e-cigarettes (87%). This knowledge was particularly limited among monolingual Mixteco speakers, only 68% of whom reported that e-cigarettes are harmful (vs. 91% of Spanish speakers, *p* <.001). Similarly, Mixteco survey respondents were least likely to know that vapes are harmful (73% vs. 90% of Spanish respondents and 100% of English respondents; *p* =.0087). Knowledge of health harms did not vary between current users and non-users.

## Discussion

Participation by Indigenous Mexican immigrants in the U.S. in large scale state and national tobacco-related research and programs has been extremely limited. As a result, information regarding tobacco use among California’s large Indigenous Mexican farmworker population is incomplete and inadequate for informing appropriate intervention development. Surveys specific to farmworker populations also lack adequate inclusion of those who speak Indigenous languages and infrequently collect information about tobacco use patterns.

To address this gap in the literature and guide tobacco program development appropriate for the agricultural worker community, we leveraged a long-standing community-academic partnership to conduct interviewer-administered surveys regarding tobacco and other products in this community [57–61]. Consistent with what is known of the Indigenous population in this region, culturally and linguistically distinct groups represented in our sample included primarily those who identify as Mixteco, followed by Zapoteco, with smaller numbers identifying as members of other Indigenous Mexican groups (e.g., Triqui, Purépecha).

While ours was a convenience sample, these data shed light on an understudied issue and demonstrate a need for interventions to prevent and control tobacco use in a historically marginalized community. Findings point to considerable use, most common among men and older individuals. Particularly notable is that rates of use were elevated among those who speak any Spanish and have been in the U.S. for some time. Reported use in this group (17%) is greater than statewide use rates of Latinos (11%), those without a high school degree (12%), those living in the highest levels of poverty (14%), and California’s general population (11%) [62]. Hence, interventions that link these community members to existing cessation tools may be an effective strategy to control community tobacco use. California’s free tobacco cessation resource, KickItCa.org, provides comprehensive cessation services via phone, text, and web in English and Spanish. Unfortunately, survey respondents by and large were not aware of publicly available prevention or cessation resources.

In contrast, little use (5%) was observed among more recent immigrants who rely exclusively on their native Indigenous language. Limited exposure to industry marketing may be one protective factor for this group; just over one-third of monolingual Mixteco speakers in our sample reported ever seeing any marketing as compared with over 40% of those in a statewide sample reporting exposure to marketing in the past week alone [62, 63]. Also of note, individuals in this group had limited knowledge of the harms of use and little exposure to prevention programming. This is not surprising given that prevention programming in both the U.S. and Mexico is most often delivered in educational settings, and the formal educational experiences of this subgroup were limited. Hence, prevention programming for this adult population, ideally early in their time in the U.S., before they become vulnerable to industry messaging in Spanish or English, could be a valuable tool in stemming future tobacco use. Such thinking is in line with well-established theories such as the Hispanic paradox and healthy immigrant effect which highlight increased health risks associated with greater time in the U.S [64, 65]. When results were examined in relation to survey language, such that the Mixteco group includes those identified as monolingual Mixteco speakers as well as those who completed the survey in Mixteco and report speaking Spanish at home, increased length of stay in the U.S. as well as greater current and ever tobacco use rates are observed in this group. These differences further underscore the need for prevention programming among those who speak only their Indigenous language.

Our data also suggest a need for community-wide educational programming related to the harms of second-hand smoke exposure, much greater in our sample than in state-level data. Over two-thirds of our sample reported exposure to tobacco smoke compared with 40% of residents reporting such exposure statewide [66]. Crowded living conditions, power dynamics associated with living in rented rooms in apartments or garages, and widespread concerns regarding involvement with authorities may exacerbate second-hand exposures. Particularly concerning are high rates of exposure reported in vehicles, given the frequency of shared transportation to work and elsewhere in the community. In line with use rates, exposure to second-hand smoke was observed least frequently among those who spoke only their native Indigenous language. Should use rates increase in this group with increased time in the U.S., so too may exposure to second-hand smoke.

While numerous participants reported that they do not allow tobacco product use in their homes, many shared that they would not ask others to refrain from use nearby even if they were bothered by the smoke/vapor. Future research to determine the best means for individuals to communicate with others regarding secondhand exposure, at home as well as in vehicles, is needed to help protect these non-users. To do this, it will be necessary to understand community knowledge related to the health harms of secondhand exposure, which was beyond the scope of our survey, but is important to address in community-level interventions.

### Limitations

The relatively small sample size, limited geographic region in which the survey was administered, and community recruitment approach used limit the extent to which our findings can be broadly generalized. Findings such as higher rates of use among older individuals should be interpreted with care given our sample included few younger individuals. Additionally, given our focus on tobacco use and concerns regarding respondent burden, we were limited in the number of items we could include regarding demographic and other characteristics. Given differences in participant language usage, language proficiency should be assessed in future work. Also, we were limited in the number of Indigenous languages we could include and unable to include those who did not speak Mixteco, Spanish or English. Members of Indigenous groups such as Zapoteco, Purepecha and Triqui in our study were Spanish or English proficient. Given differences observed between monolingual Mixteco speakers and those who spoke any Spanish, it will be important to consider the tobacco needs of other monolingual Indigenous language speakers in future work.

### New Contribution to the Literature and Conclusions

This work contributes to the currently limited health-related literature regarding Indigenous Mexican agricultural workers, a historically marginalized population that has to date been underrepresented in health research, including with respect to tobacco use. Results point to a number of unmet needs in this community. Specifically, findings from this cross-sectional survey demonstrate a need for tailored tobacco prevention programming, particularly among more recent immigrants who primarily rely on their native Indigenous language. Linkages to cessation resources may be especially important for those who have been in the U.S. longer and have Spanish proficiency. These results are guiding the next steps in our program of community-academic partnered research aimed at developing, assessing and disseminating tailored anti-tobacco programming for this historically marginalized population, state and nationwide.

## Data Availability

Data collected from community partners are maintained by the study team. Oral consent was obtained to use these data for the express puposes of this community-academic research project.
