# Correction: Tobacco Use, Experiences and Knowledge among Indigenous Mexican Agricultural Workers

**DOI:** 10.1007/s10903-026-01852-9

**Published:** 2026-01-17

**Authors:** Alison K. Herrmann, Genevieve Flores-Haro, Barbara Berman, Alison M. Elliott, Maritza Lopez, L. Cindy Chang, Norma Gonzalez, Catherine M. Crespi, Michael K. Ong, Arcenio Lopez, Roshan Bastani

**Affiliations:** 1 School of Public Health, UCLA Kaiser Permanente Center for Health Equity UCLA Fielding University of California, Los Angeles, Los Angeles, USA; 2 Jonsson Comprehensive Cancer Center, University of California, Los Angeles, Los Angeles, USA; 3 Mixteco Indígena Community Organizing Project, Oxnard, USA; 4 School of Medicine, University of California, Los Angeles, Los Angeles, USA; 5 VA Greater Los Angeles Healthcare System, Los Angeles, USA

**Correction to: Journal of Immigrant and Minority Health.**

10.1007/s10903-025-01840-5.

The original version of this article contained an error in co-author name.

The ninth author name should have been Michael K. Ong instead of Micheal K. Ong.

The original article has been corrected.

